# Nuclear Hormone Receptor Expression in Mouse Kidney and Renal Cell Lines

**DOI:** 10.1371/journal.pone.0085594

**Published:** 2014-01-22

**Authors:** Daisuke Ogawa, Jun Eguchi, Jun Wada, Naoto Terami, Takashi Hatanaka, Hiromi Tachibana, Atsuko Nakatsuka, Chikage Sato Horiguchi, Naoko Nishii, Hirofumi Makino

**Affiliations:** 1 Department of Medicine and Clinical Science, Okayama University Graduate School of Medicine, Dentistry and Pharmaceutical Sciences, Okayama, Japan; 2 Department of Diabetic Nephropathy, Okayama University Graduate School of Medicine, Dentistry and Pharmaceutical Sciences, Okayama, Japan; Baylor College of Medicine, United States of America

## Abstract

Nuclear hormone receptors (NHRs) are transcription factors that regulate carbohydrate and lipid metabolism, immune responses, and inflammation. Although several NHRs, including peroxisome proliferator-activated receptor-γ (PPARγ) and PPARα, demonstrate a renoprotective effect in the context of diabetic nephropathy (DN), the expression and role of other NHRs in the kidney are still unrecognized. To investigate potential roles of NHRs in the biology of the kidney, we used quantitative real-time polymerase chain reaction to profile the expression of all 49 members of the mouse NHR superfamily in mouse kidney tissue (C57BL/6 and db/m), and cell lines of mesangial (MES13), podocyte (MPC), proximal tubular epithelial (mProx24) and collecting duct (mIMCD3) origins in both normal and high-glucose conditions. In C57BL/6 mouse kidney cells, hepatocyte nuclear factor 4α, chicken ovalbumin upstream promoter transcription factor II (COUP-TFII) and COUP-TFIII were highly expressed. During hyperglycemia, the expression of the NHR 4A subgroup including neuron-derived clone 77 (Nur77), nuclear receptor-related factor 1, and neuron-derived orphan receptor 1 significantly increased in diabetic C57BL/6 and db/db mice. In renal cell lines, PPARδ was highly expressed in mesangial and proximal tubular epithelial cells, while COUP-TFs were highly expressed in podocytes, proximal tubular epithelial cells, and collecting duct cells. High-glucose conditions increased the expression of Nur77 in mesangial and collecting duct cells, and liver x receptor α in podocytes. These data demonstrate NHR expression in mouse kidney cells and cultured renal cell lines and suggest potential therapeutic targets in the kidney for the treatment of DN.

## Introduction

Diabetic nephropathy (DN) is a major microvascular complication in patients with diabetes mellitus, ultimately leading to end-stage renal diseases [1]. The incidence of DN is increasing rapidly with the increase in patients with type 2 diabetes and metabolic syndrome, and at present accounts for almost 50% of all end-stage renal diseases [2]. It is characterized by the accumulation of extracellular matrix in the glomerular and tubulointerstitial compartments and by the thickening and hyalinization of intrarenal vasculature. Various pathogenic mechanisms of DN have been proposed including increased expression of advanced glycation end-products, protein kinase C, transforming growth factor β, and reactive oxygen species. In addition to these metabolic derangements, changes in the glomerular hemodynamics, modulated in part by local activation of the renin-angiotensin system, synergistically exacerbate the progression of DN. Despite notable advances in the treatment of diabetes mellitus, current therapies do not fully suppress the incidence of DN. Therefore, identification of additional causative factors leading to renal injury and the development of novel agents to prevent or treat DN are urgently needed.

In humans, 48 members of the nuclear hormone receptor (NHR) superfamily of transcription factors have been identified (49 in mice). Several modulators of NHRs have been developed as oral drugs for the treatment of diabetes and dyslipidemia. Synthetic agonists for peroxisome proliferator-activated receptor-γ (PPARγ) and PPARα, such as thiazolidinediones and fibrates, improve glycemic control in type 2 diabetic patients and lower serum triglyceride levels in hyperlipidemic patients [3]. Moreover, these drugs are reported to have protective effects against renal dysfunction [4], [5], [6]. However, no drugs that modulate NHRs, other than PPARγ and PPARα agonists, have been released in the market. In animal models of DN, several agonists of NHRs, including the vitamin D receptor (VDR) [7], [8], the farnesoid X receptor (FXR) [9], and the estrogen receptor (ER) [10] have been reported to have potential suppressive effects on the progression of DN. We have also demonstrated that synthetic agonists of PPARδ [11] and the liver X receptor (LXR) [12] reduce urinary albumin excretion in a mouse model of DN. Moreover, hepatocyte nuclear factor α (HNF4α) [13] and estrogen-related receptor (ERR) [14] are also thought to have a role in the pathogenesis of DN.

There is increasing evidence of NHR expression in peripheral tissues including adipose tissue [15], macrophages [16], and endocrine pancreas tissue [17], but little is known about the expression and function of NHRs in the kidney. To elucidate their potential role in the pathogenesis of DN, we have performed a comprehensive analysis of NHR expression in mouse kidney tissue and renal cell lines.

## Materials and Methods

### Animals

Eight-week-old male C57BL/6J mice were purchased from Charles River (Yokohama, Japan). Diabetes was induced by peritoneal injection of 200 mg/kg streptozotocin (Sigma-Aldrich, Tokyo, Japan) in citrate buffer (pH 4.5). C57BL/6J mice were euthanized at 8 weeks after the induction of diabetes. Six-week-old male diabetic *db/db* mice (BKS.Cg-*lepr^db^/lepr^db^*) and male non-diabetic *db/m* mice (BKS.Cg-*lepr^db^/*+) were purchased from CLEA Japan (Tokyo, Japan). *Db/db* mice and *db/m* mice were euthanized at 9 weeks of age. All mice were maintained under a 12-h light/12-h dark cycle with free access to food and tap water. Animal care and procedures were performed according to the Guidelines for Animal Experimentation at Okayama University, the Japanese Government Animal Protection and Management Law, and the Japanese Government Notification on Feeding and Safekeeping of Animals. The experimental protocol was approved by the Animal Ethics Review Committee of Okayama University (OKU-2011326). All surgery was performed under sodium pentobarbital anesthesia, and every effort was made to minimize suffering.

### Cell culture

Murine mesangial (MES13), podocyte (MPC), proximal tubular epithelial (mProx24), and collecting duct (mIMCD3) cells were cultured as previously described [18], [19], [20]. For high-glucose stimulation, renal cell lines were serum-starved in 0.5% fetal bovine serum for 24 h. Subsequently, all cells were exposed to low-glucose (5.5 mM) or high-glucose (25 mM) conditions for 24 h before RNA isolation.

### RNA measurement

RNA was isolated from kidney cortex samples or cultured cells using an RNeasy Mini kit (Qiagen, Valencia, CA, USA). Single-strand cDNA was synthesized from the extracted RNA using a real-time polymerase chain reaction (RT-PCR) kit (Perkin Elmer, Foster City, CA, USA). To evaluate the mRNA expression of each NHR, quantitative RT-PCR (qPCR) was performed using TaqMan® Array Plates and StepOnePlus™ (Applied Biosystems, Foster City, CA, USA) and TaqMan® Fast Universal PCR Master Mix (Applied Biosystems). Primers were purchased from Applied Biosystems. Each sample was analyzed in quadruplicate and normalized for S18 mRNA expression. Primer sequences for mouse genes are provided in Table S1.

### Immunohistochemistry

Immunofluorescent staining was performed as described previously [21]. The expression of VDR in cultured renal cells was detected using rat anti-VDR antibody (Abcam, Cambridge, UK) followed by Alexa Fluor 488 goat anti-rat IgG (Invitrogen, Carlsbad, CA). Similarly, PPARδ and COUP-TFII were detected using rabbit anti-PPARδ (Affinity Bioreagents, Golden, CO) and anti-COUP-TFII antibody (Abcam) followed by Alexa Fluor 488 goat anti-rabbit IgG (Invitrogen). Renal expression of NOR1 was detected using mouse anti-NOR1 antibody (Abcam) followed by Alexa Fluor 488 goat anti-mouse IgG (Invitrogen). To determine whether NOR1 was localized in mesangial cells, podocytes, proximal tubular epithelial cells, or collecting duct cells, the sections were counter-stained with rabbit anti-fibronectin antibody (Sigma-Aldrich, St. Louis, MO), rabbit anti-WT-1 antibody (Abcam, Cambridge, UK), rabbit anti-aquaporin 1 (AQP1) antibody (Millipore, Temecula, CA), or rabbit anti-AQP2 antibody (Abcam) respectively, followed by Alexa Fluor 594 goat anti-rabbit IgG (Invitrogen). Fluorescence images were obtained using a fluorescence microscope (BX51; Olympus, Tokyo, Japan).

## Results

The expression of NHRs in mouse kidney tissue (C57/BL6 and db/m) and renal (mesangial, podocyte, proximal tubular epithelial, and collecting duct) cell lines was determined by qPCR analysis.

**Figure 1:**
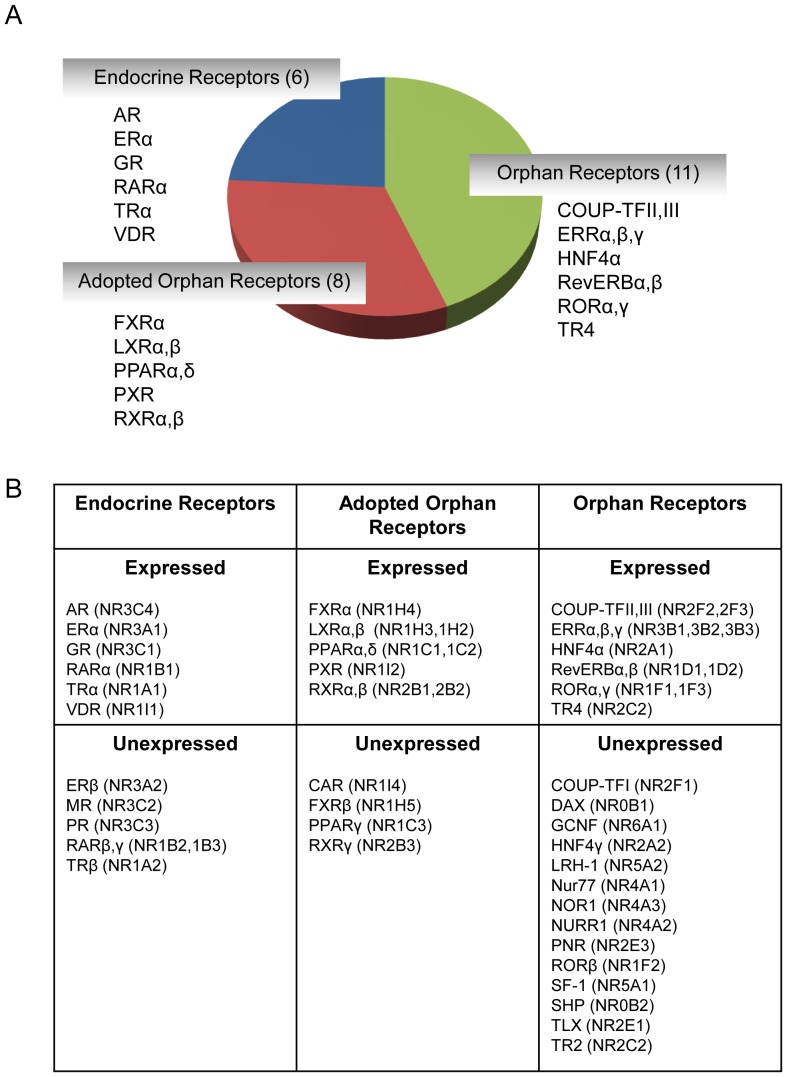
Composition of nuclear hormone receptors (NHRs) in the kidney of C57BL/6 mice. (A) Twenty-five of 49 known NHRs are expressed in C57BL/6 mouse kidney. These include six endocrine receptors that bind hormonal lipids with high-affinity, eight adopted orphan receptors that bind dietary lipids with low-affinity, and 11 orphan receptors. Constituent receptors of each of these classes are listed. (B) Tabular listing of NHRs expressed or unexpressed in C57BL/6 mouse kidney along with their classification and nomenclature. Receptors were deemed unexpressed if cycle threshold (Ct) values exceeded 31.

**Figure 2:**
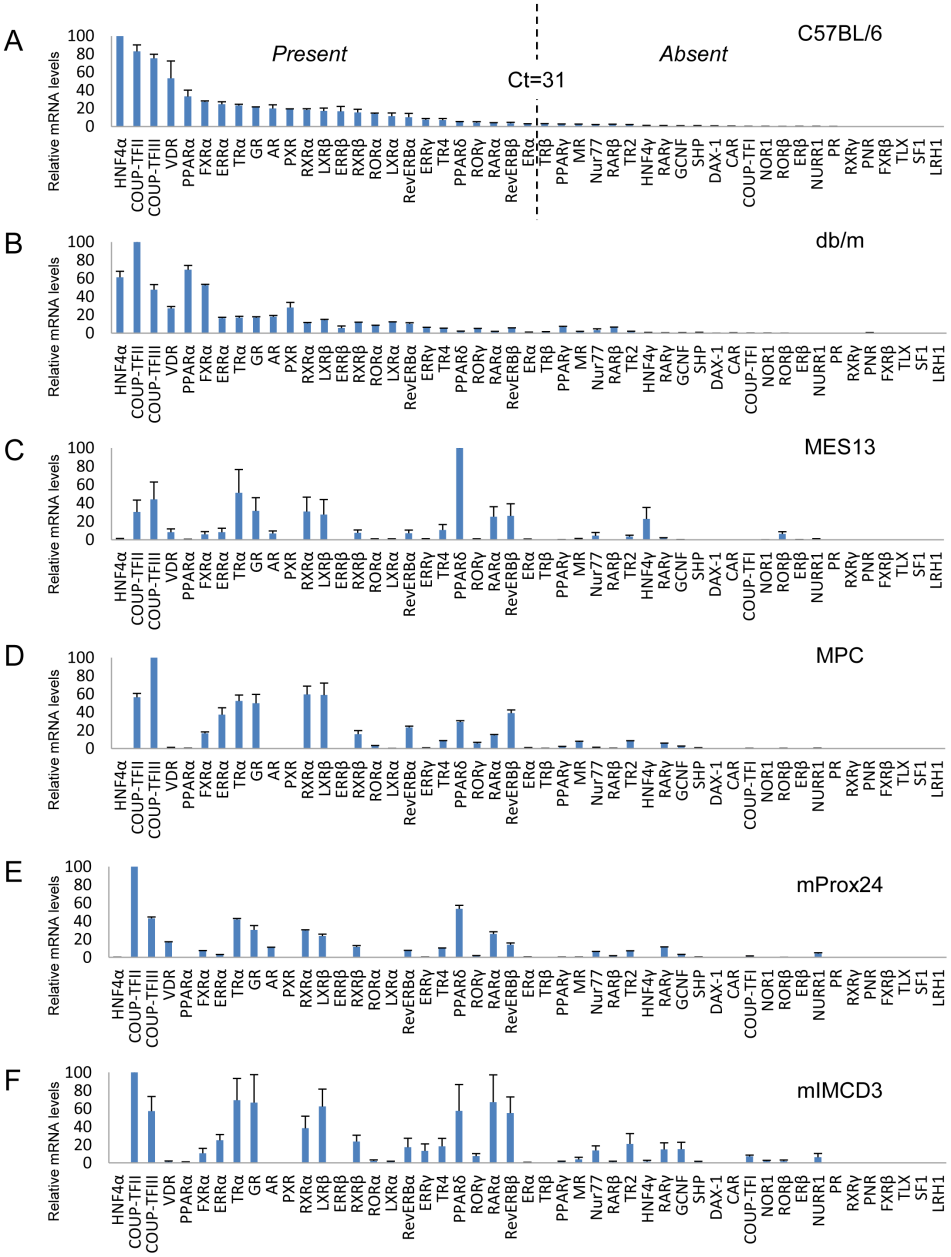
Comparative expression levels of 49 nuclear hormone receptors (NHRs) in mouse kidney tissue and renal cell lines. The relative mRNA levels are depicted for mouse kidney (C57BL/6 (A) and db/m (B)) and mesangial (MES13) (C), podocyte (MPC) (D), proximal tubular epithelial (mProx24) (E) and collecting duct (mIMCD3) (F) cell lines. All values are expressed relative to 18S and arithmetically adjusted to depict the highest-expressed NHR for each tissue/cell line as a unit of 100. Values represent the means ± SEM of three independent samples of each tissue or cell line. Setting arbitrary cutoffs at Ct<31 (present) or Ct>31 (absent), as shown by broken lines in the C57BL/6 mouse kidney panel, reveals that 25 NHRs were expressed and six NHRs were not detected in C57BL/6 mouse kidney.

**Figure 3:**
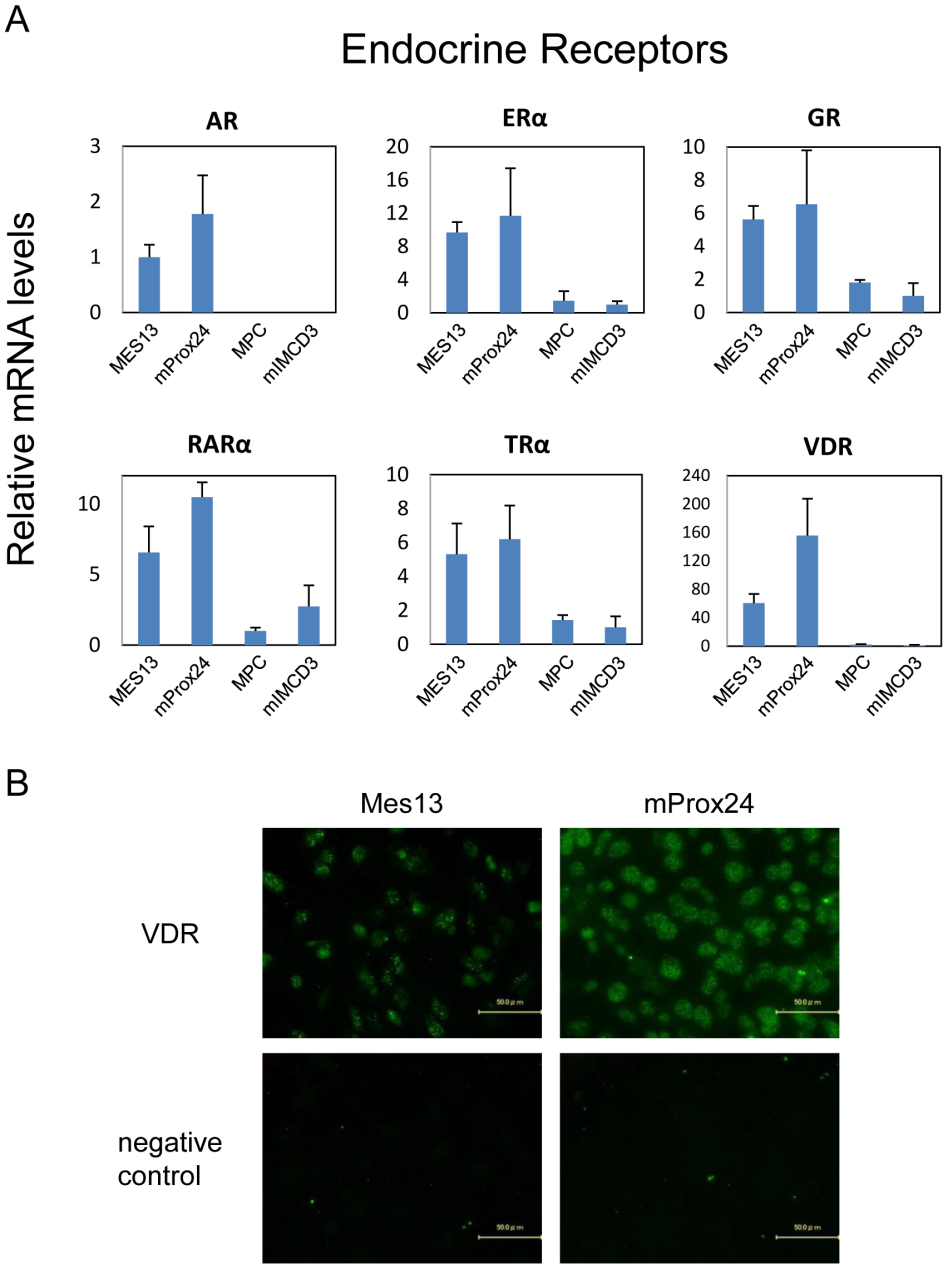
Endocrine receptors expressed in renal cell lines. (A) The relative mRNA levels are depicted for mesangial (MES13), podocyte (MPC), proximal tubular epithelial (mProx24), and collecting duct (mIMCD3) cell lines. All values are expressed relative to 18S and arithmetically adjusted to depict the lowest-expressing sample as a unit of 1. Values represent the means ± SEM of three independent samples of each cell lines, and the results are representative of two independent studies. (B) Representative photomicrographs of immunofluorescent staining. Vitamin D receptor (VDR) was predominantly expressed in mProx24 cells, and to a lesser extent in MES13 cells.

**Figure 4:**
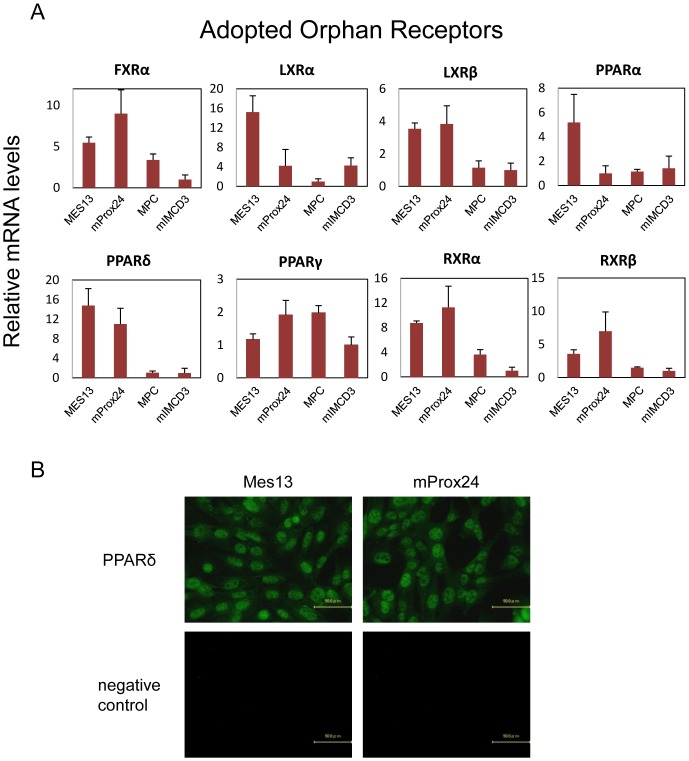
Adopted orphan receptors expressed in renal cell lines. (A) Refer to the legend for Figure 3A for details. (B) Representative photomicrographs of immunofluorescent staining. Peroxisome proliferator-activated receptor-δ (PPARδ) was expressed in both MES13 and mProx24 cell lines.

**Figure 5:**
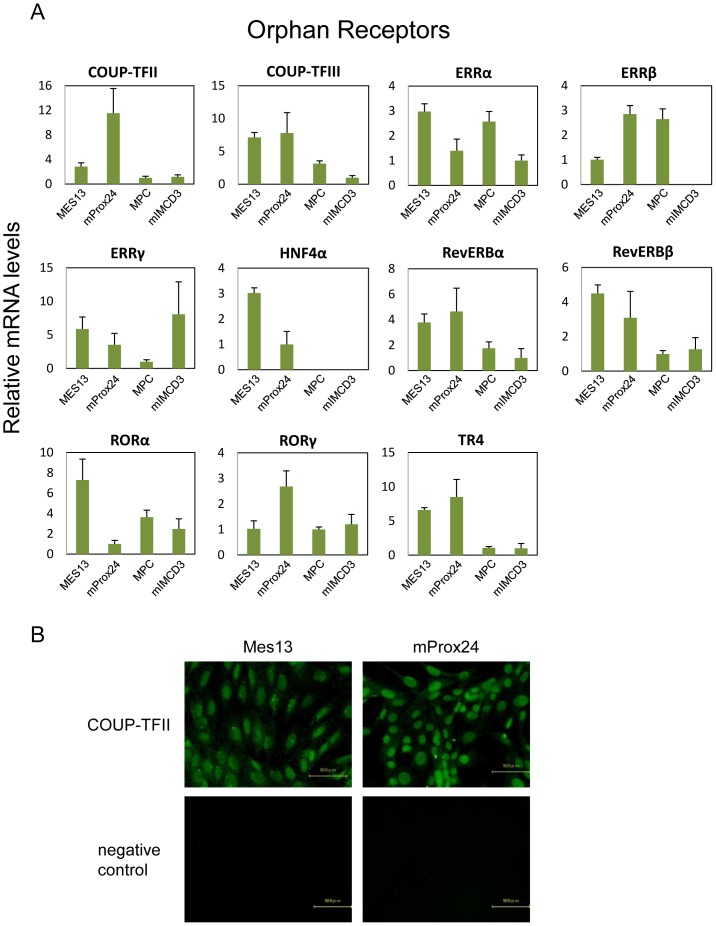
Orphan receptors expressed in renal cell lines. (A) Refer to the legend for Figure 3A for details. (B) Representative photomicrographs of immunofluorescent staining. Chicken ovalbumin upstream promoter transcription factor II (COUP-TFII) was expressed in both MES13 and mProx24 cell lines.

**Figure 6:**
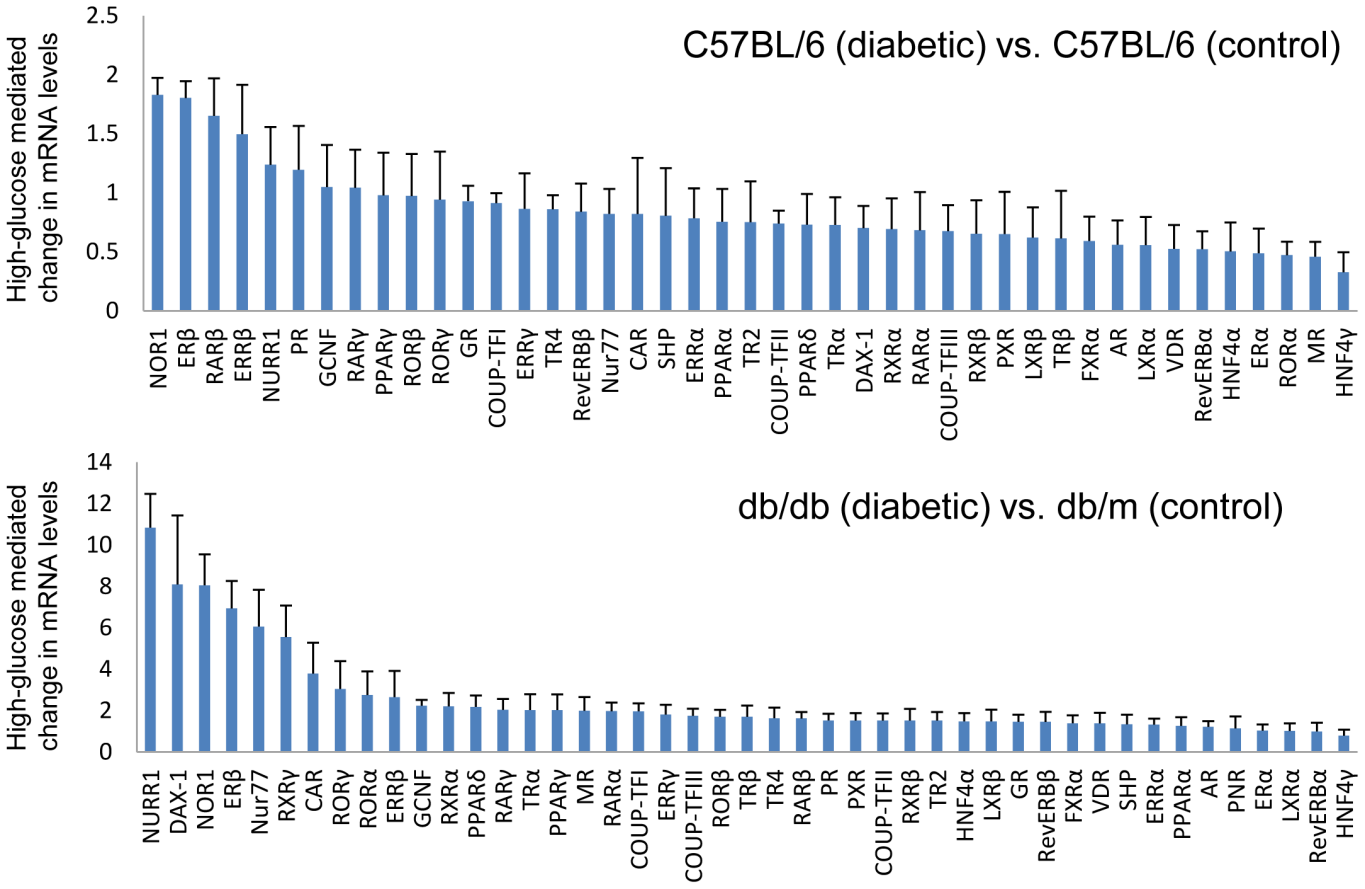
Comparative expression levels of NHRs in mouse kidney tissue under high-glucose conditions. The relative mRNA levels are depicted for streptozotocin-induced diabetic C57BL/6 mouse kidney compared with control C57BL/6 mouse kidney (upper panel), and diabetic db/db mouse kidney compared with control db/m mouse kidney (lower panel). Values depict the means ± SEM of three independent samples.

**Figure 7:**
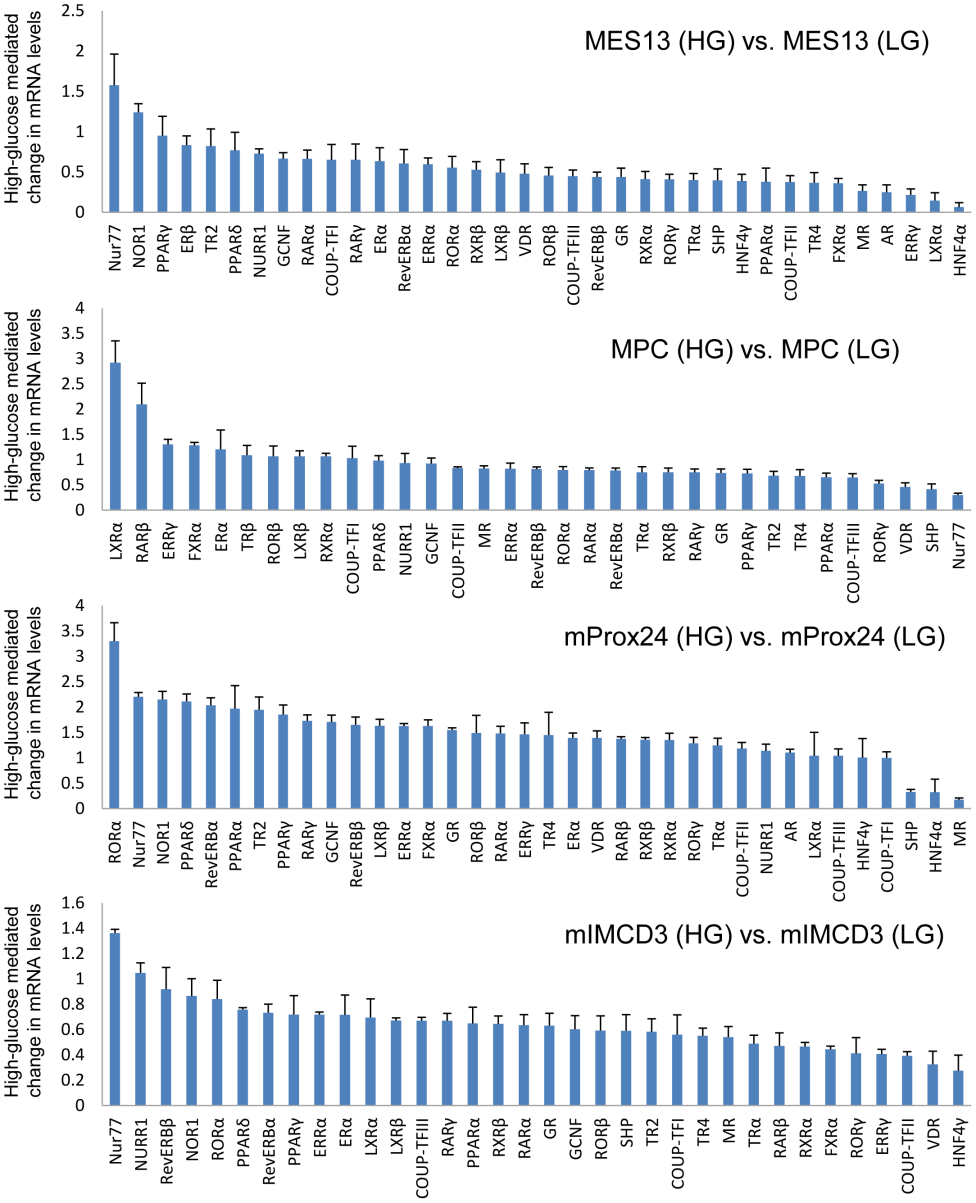
Comparative expression levels of NHRs in renal cell lines under high-glucose conditions. Mesangial (MES13), podocyte (MPC), proximal tubular epithelial (mProx24), and collecting duct (mIMCD3) cell lines. Each panel displays NHR expression under high-glucose conditions compared with low-glucose conditions. Values depict the means ± SEM of three independent samples. HG, high-glucose. LG, low-glucose.

**Figure 8:**
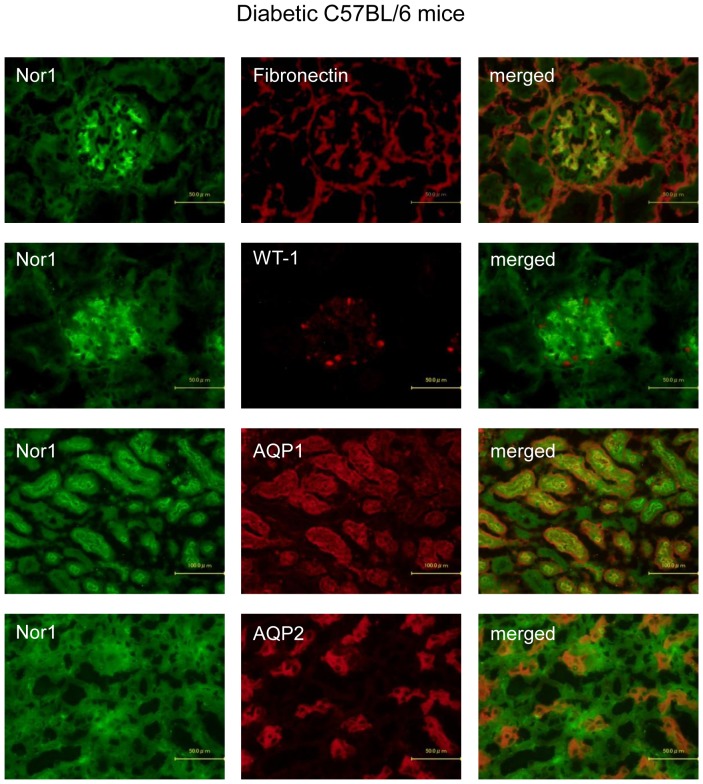
Representative photomicrographs of double immunofluorescent staining in diabetic C57BL/6 mice. NOR1 expression was localized in mesangial, proximal tubular epithelial, and collecting duct cells, but not in podocytes in the kidneys of diabetic C57BL/6 mice.

**Figure 9:**
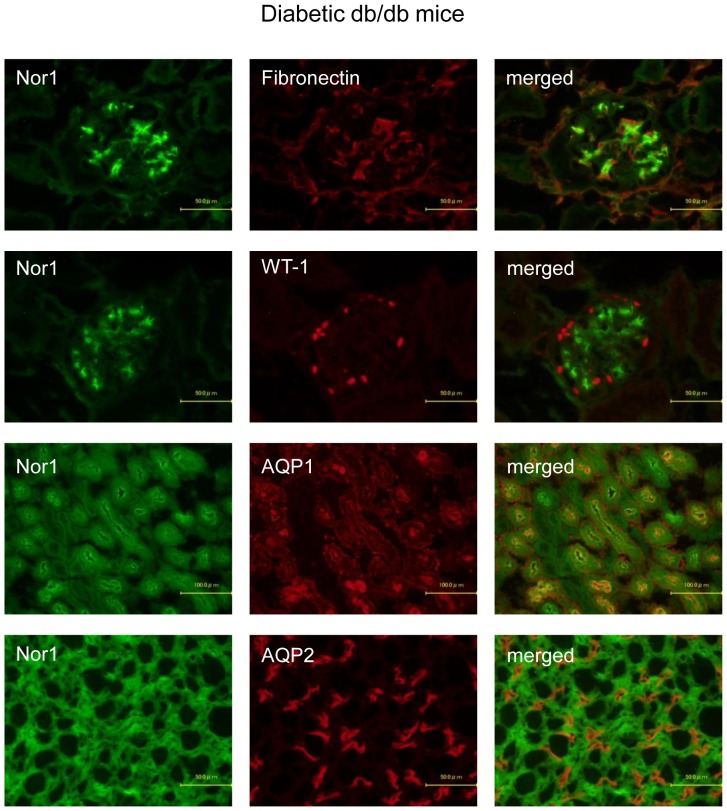
Representative photomicrographs of double immunofluorescent staining in diabetic db/db mice. NOR1 expression was localized in mesangial, proximal tubular epithelial, and collecting duct cells, but not in podocytes in the kidneys of diabetic db/db mice.

The composition and rank order of NHR expression in C57/BL6 mouse kidney are shown in Figures 1 and 2A, respectively. We then analyzed NHR expression in db/m mice and renal cell lines (Figs. 2B–F, 3, 4, and 5). This format is consistent with previous NHR expression analyses and provides a basis for comparison with cell lines commonly associated with each receptor type. Furthermore, because this survey focused on expression in a select few cell types (mouse kidney, renal cell lines), we could analyze RNA levels for all receptors in a single assay for direct comparison of NHR levels within a given tissue. Finally, we elucidated the effect of elevated glucose on NHR expression in mouse kidney and renal cell lines (Fig. 6, 7, 8, and 9).

### NHR expression in kidney

The composition of NHR expression in C57/BL6 mouse kidney is shown in Figure 1. Receptors were deemed to be expressed if cycle threshold (Ct) values were less than 31. Composite gene expression analysis of the NHR superfamily revealed the presence of 25 of the 49 known NHRs in C57BL/6 mouse kidney. These included six members of the endocrine receptor family, which are activated by high-affinity hormonal lipids, eight adopted heterodimeric orphan receptors, which are regulated by low-affinity dietary lipids, and 11 true orphan receptors (Fig. 1A). Figure 1B shows a complete tabulation of the expressed and unexpressed receptors along with their classification and nomenclature [22]. Six NHRs including FXRβ, liver receptor homolog-1 (LRH1), photoreceptor cell-specific nuclear receptor (PNR), retinoid X receptor γ (RXRγ), steroidogenic factor 1 (SF1), and Tailles homolog orphan receptor (TLX) were not detected in normal C57BL/6 mouse kidney.

### Rank order of NHR expression in mouse kidney tissue and renal cell lines

The relative mRNA levels of NHRs expressed in C57BL/6 mouse kidney are shown in rank order in Figure 2A. For the purposes of comparison, similar analyses were conducted on db/m mouse kidney and renal cell lines (mesangial cell, MES13; podocytes, MPC; proximal tubular epithelial, mProx24, and collecting duct, mIMCD3) (Fig. 2B–F).

In C57BL/6 mouse kidney, the most abundant receptor was HNF4α and other abundant receptors were chicken ovalbumin upstream promoter transcription factor II (COUP-TFII) and COUP-TFIII, VDR, PPARα, FXRβ, and ERRα. In contrast, COUP-TFII was the most abundant NHR in db/m mouse kidney, and the mRNA levels of HNF4α, COUP-TFIII, and VDR were lower in db/m mice than in C57BL/6 mice.

A similar survey of NHR transcripts in renal cell lines revealed significant differences compared with the receptor expression pattern observed in mouse kidney. COUP-TFII was the most abundant NHR in the mProx24 and mIMCD3 cell lines, and the second most abundant receptor in the MPC cell line. In contrast, PPARδ was the most highly expressed receptor in the MES13 cell line, and the second most abundant NHR in the mProx24 cell line. In addition to PPARδ, thyroid hormone receptor α (TRα), COUP-TFII, COUP-TFIII, glucocorticoid receptor (GR), RXRα, and LXRβ were found to be abundant receptors in the MES13 cell line. In the MPC cell line, COUP-TFII, COUP-TFIII, RXRα, LXRβ, TRα, and GR were highly expressed, while COUP-TFII, PPARδ, COUP-TFIII, and TRα were abundant in the mProx24 cell line. In the mIMCD3 cell line, the expression pattern of NHRs was similar to that in the MPC cell line. Furthermore, retinoic acid receptor α (RARα), reverse-ErbAβ (Rev-ERBβ), and PPARδ were highly expressed NHRs in the mIMCD3 cell line.

### Endocrine receptors in renal cell lines

The expression of endocrine receptors was compared for all renal cell lines (Fig. 3A). Androgen receptor (AR), RARα, and VDR were highly expressed NHRs in the mProx24 cell line. In contrast, AR and VDR were hardly expressed in the MPC and mIMCD3 cell lines. ERα, GR, and TRα were abundant in both the mProx24 and MES13 cell lines. Immunohistochemistry of VDR showed that the intensity of VDR was higher in the mProx24 cell line than in the MES13 cell line (Fig. 3B).

### Adopted orphan receptor expression in renal cell lines

The expression of adopted orphan receptors in the renal cell lines was also compared (Fig. 4A). LXRα and PPARα were highly expressed in the MES13 cell line, while LXRβ and PPARδ were highly expressed in both the MES13 and mProx24 cell lines. FXRα, RXRα, and RXRβ were predominantly expressed in the mProx24 cell line. In contrast, adopted orphan receptors were expressed at low levels in the MPC and mIMCD3 cell lines. Immunofluorescent staining showed that PPARδ was expressed in both the MES13 and mProx24 cell lines (Fig. 4B).

### Orphan receptor expression in renal cell lines

We also compared the expression of orphan receptors in the renal cell lines (Fig. 5A). ERRα, HNF4α, retinoic acid-related orphan receptor α (RORα), and RevERBβ were highly expressed in the MES13 cell line, while COUP-TFII and RORγ were highly expressed in the mProx24 cell line. COUP-TFIII, RevERBα, and testicular orphan receptor 4 (TR4) were abundant in both the mProx24 and MES13 cell lines. Intriguingly, high expression of ERRα was detected in the MPC cell line, while ERRγ predominated in the mIMCD3 cell line. Immunohistochemistry showed that COUP-TFII was expressed in both the MES13 and mProx24 cell lines (Fig. 5B).

### Glucose regulation of NHR expression in mouse kidney tissue and renal cell lines

To determine differential expression of NHRs in response to hyperglycemia, we compared the expression of NHRs in the kidneys of streptozotocin-induced type 1 diabetic C57BL/6 mice and control C57BL/6 mice (Fig. 6). Similarly, we compared NHR expression in the kidneys of type 2 diabetic db/db mice and control db/m mice (Fig. 6). Furthermore, we evaluated the expression of the 49 NHRs in renal cell lines to determine whether changes in RNA levels occur in high-glucose conditions (Fig. 7). Renal cell lines were exposed to low-glucose (5.5 mM) or high-glucose (25 mM) conditions for 24 h before RNA isolation.

Significantly increased RNA levels were observed for neuron-derived orphan receptor 1 (NOR1) and nuclear receptor-related factor 1 (NURR1), members of the NHR 4A (NR4A) subgroup, and ERβ and ERRβ, members of the NR3A subgroup, in the kidneys of streptozotocin-induced diabetic C57BL/6 mice (Fig. 6). A similar increase in NURR1, NOR1, and ERβ mRNA was observed in the kidneys of diabetic db/db mice (Fig. 6). Interestingly, neuron-derived clone 77 (Nur77), which is a member of the NR4A subgroup, was also upregulated as well as NURR1 and NOR1 in db/db mouse kidney.

A similar evaluation of glucose-mediated changes in gene expression in renal cell lines was performed (Fig. 7). In the MES13 cell line, upregulation of the expression of the NR4A members of the NHR superfamily (Nur77>NOR1>NURR1), as well as that of PPARγ and PPARδ was detected. The rank order of NHR expression in the MPC cell line was LXRα, RARβ, ERRβ, FXRα, ERα, TRβ, RORβ, LXRβ. In the mProx24 cell line, RORα was the most highly upregulated receptor while Nur77, NOR1, PPARδ, RevERBα, and PPARα were also upregulated in high-glucose conditions. Similar to the MES13 cell line, high-glucose exposure increased the expression of the NR4A members (Nur77>NURR1>NOR1) as well as RevERBβ, RORα, PPARδ, and RevERBα in the mIMCD3 cell line.

Since the RNA level of NOR1 was the highest in the kidneys of streptozotocin-induced diabetic C57BL/6 mice, and the third highest in diabetic db/db mice (Fig. 6), we confirmed NOR1 expression by immunohistochemistry. The expression of NOR1 in the glomeruli of diabetic C57BL/6 mice coexists with fibronectin, a marker for mesangial cells, but not with WT-1, a marker for podocytes (Fig. 8). On the other hand, NOR1 expression in the interstitium of diabetic C57BL/6 mice coexists with both AQP1 and AQP2, markers for proximal tubular epithelial and collecting duct cells, respectively (Fig. 8). These expressions are consistent with those in qPCR (Fig. 7). Similarly, NOR1 expression was detected in mesangial, proximal tubular epithelial, and collecting duct cells, but not in podocytes in the kidneys of diabetic db/db mice (Fig. 9).

## Discussion

NHRs are one of the largest transcription factor families in the mammalian genome. A total of 48 human and 49 mouse genes that encode NHRs have been identified [23]. Recently, the quantitative assessment of NHR RNA levels has been established for various mouse tissues including adipocytes [15], macrophages [16], pancreas [17], [24], retina [25], and during the circadian cycle in mouse liver, adipose, and muscle [26] by using qPCR. However, the expression of NHRs in the kidney remains unknown. Therefore, we analyzed NHR expression in mouse kidney tissue and renal cell lines.

In this study, we identified 25 NHRs that are expressed in C57BL/6 mouse kidney. The receptors detected in abundance, including HNF4α, VDR, PPARα, FXR, and ERRα, have already been reported to play a role in the pathogenesis of DN [27]. However, we provide the first evidence that COUP-TFII and COUP-TFIII are expressed abundantly in C57BL/6 mouse kidney, with COUP-TFII being the most abundant NHR in the kidneys of db/m mice. In the renal cell lines, COUP-TFII was shown to be the most abundant NHR in proximal tubular epithelial and collecting duct cells, and highly expressed in mesangial cells and podocytes. Similarly, COUP-TFIII was shown to be the most abundant NHR in podocytes, and highly expressed in mesangial cells, proximal tubular epithelial cells, and podocytes. COUP-TFII is thought to have a role in kidney development and to be necessary for metanephric mesenchyme formation and kidney precursor cell survival [28], [29]. In contrast, the role of COUP-TFII in DN is completely unknown and further studies are required.

The analysis of NHR expression in renal cell lines showed that most are expressed mainly in proximal tubular epithelial cells and mesangial cells. However, we showed that PPARα and LXRα are more highly expressed in mesangial cells than in the other renal cell types, which is consistent with previous reports that PPARα and LXRα are expressed in mesangial cells [30], [31], [32], [33]. Since synthetic agonists for PPARα and LXRα are reported to increase cholesterol efflux and ameliorate lipid-related glomerular disease [31], [32], [34], it can be speculated that these NHRs in mesangial cells represent therapeutic targets for treating glomerular injuries in DN. Moreover, the endocrine receptors, ERα, TRα, and VDR, the adopted orphan receptors, PPARδ, LXRβ, and FXRα, and the orphan receptors, COUP-TFII, COUP-TFIII, RORα, RORγ, RevERBα, and RevERBβ, are expressed not only in mesangial cells, but also in proximal tubular epithelial cells. Furthermore, the expressions of endocrine receptor VDR, orphan receptor PPARδ, and adopted orphan receptor COUP-TFIII were confirmed by immunohistochemistry (Figs. 3B, 4B, 5B). RevERBs and RORs are thought to play important roles in the regulation of circadian rhythm [26]; however, their functions in the kidney remain to be fully elucidated. Our data suggest that these receptors play fundamental roles in renal function.

ERRs are highly expressed in the kidney and renal cell lines. Intriguingly, ERRα is the only NHR that is highly expressed in podocytes, while ERRγ is the only NHR that is highly expressed in collecting duct cells. It is reported that ERRα is a key regulator of interferon-γ-induced mitochondrial reactive oxygen species production and host defense [35], while both ERRα and ERRγ synergistically orchestrate a comprehensive cardiac transcriptional program [36]. Regarding the kidney, ERRα and ERRγ are expressed in the outer stripe of the outer medulla of mouse kidney [37], suggesting that these receptors regulate renal potassium homeostasis and the renin-angiotensin system [38], [39]. Although ERRα is induced by PPARγ coactivator-1α (PGC-1α) [40], little is known about the role and function of ERRα in podocytes and further studies are needed.

The most interesting and novel finding in this study is that the members of the NR4A subgroup, including NOR1, NURR1, and Nur77, are highly induced in the kidneys of diabetic C57BL/6 and db/db mice. All three members of the NR4A subgroup are expressed in energy-dependent tissues such as skeletal muscle, brain, adipose tissues, heart, and liver suggesting a possible role in energy metabolism [41]. Although it is known that kidney expression of the NR4A subgroup is upregulated by stimulation of parathyroid hormone [42] or prostaglandin E2 [43], the effects of high-glucose stimulation on the expression of these receptors have not been reported. To confirm the results of qPCR, we performed immunohistochemistry of NOR1 in the kidneys of diabetic C57BL/6J and db/db mice. Double staining of NOR1 with markers for mesangial cells (fibronectin), podocytes (WT-1), proximal tubular cells (AQP1), and collecting duct cells (AQP2) revealed that NOR1 was expressed in mesangial cells, proximal tubular cells, and collecting duct cells, but not in podocytes (Fig. 8 and 9). Our in vitro studies showed that NOR1, Nur77, and NURR1 are also highly upregulated in cultured mesangial cells, proximal tubular epithelial cells, and collecting duct cells. These results might help to elucidate the previously unrecognized mechanisms of the progression of DN. To the best of our knowledge, there have been no studies investigating the expression of NR4A subgroup in patients with DN, and further studies using renal biopsy specimens are needed as the next step.

We were surprised that the expression profile of NHRs in the kidneys of diabetic C57BL/6J and db/db mice is partially overlapping like the members of the NR4A subgroup, but mostly different as shown in Fig. 6. This difference may be caused by the effect of streptozotocin injection in C57BL/6J mice or by deletion of the leptin receptor gene in db/db mice. Another surprise was that these NHRs were not differentially upregulated or even downregulated in the kidneys of diabetic mice or high-glucose treated renal cell lines. Several agonists of NHRs, including PPARγ, PPARα, VDR, and FXR have been reported to have potential suppressive effects on the progression of DN [44]. Our results are consistent with the reports of previous studies that the expression of PPARα, VDR, and FXR was downregulated by the induction of diabetes [33], [45]. The precise mechanism for downregulation of these NHRs in response to hyperglycemia is unknown, but activation of these NHRs may prevent the development of DN in mouse models. Based on these observations, we speculate that the inactivation of upregulated NHRs in hyperglycemia, such as NOR1, NURR1, and Nur77, may also be a potential therapeutic approach in the management of DN. Further studies are required to address whether modulation of these NHRs will provide a novel strategy for treating DN.

In summary, we have analyzed the expression of all NHRs present in normal and high-glucose conditions in mouse kidney tissue and renal cell lines. The functions of most of these NHRs are still unknown; however, our data provide the basis for further studies to elucidate potential therapeutic targets in the pathogenesis of DN.

## Supporting Information

Table S1 **Primers used for quantitative real-time PCR.**
(DOC)
