# Supplementary material for: Nuclear Hormone Receptor Expression in Mouse Kidney and Renal Cell Lines
Source: PLoS One. 2014 Jan 22;9(1):e85594. doi: 10.1371/journal.pone.0085594 (PMC3899020; doi:10.1371/journal.pone.0085594)
Supplement: Table S1 — Primers used for quantitative real-time PCR. (DOC) [file pone.0085594.s001.doc]

**Supporting Information**

**Table S1.**

| Gene abbreviation | Gene name | Accession number | Assay ID |
| --- | --- | --- | --- |
| NR1A1 | TRα | NM_178060 | Mm00617505_m1 |
| NR1A2 | TRβ | NM_009380 | Mm00437044_m1 |
| NR1B1 | RARα | NM_009024 | Mm00436264_m1 |
| NR1B2 | RARβ | NM_011243 | Mm01319680_m1 |
| NR1B3 | RARγ | NM_011244 | Mm00441083_m1 |
| NR1C1 | PPARα | NM_011144 | Mm00440939_m1 |
| NR1C2 | PPARδ | NM_011145 | Mm00803186_g1 |
| NR1C3 | PPARγ | NM_011146 | Mm00440945_m1 |
| NR1D1 | RevERBα | NM_145434 | Mm00520708_m1 |
| NR1D2 | RevERBβ | NM_011584 | Mm00441730_m1 |
| NR1F1 | RORα | NM_013646 | Mm00443103_m1 |
| NR1F2 | RORβ | NM_146095 | Mm00524993_m1 |
| NR1F3 | RORγ | NM_011281 | Mm00441144_g1 |
| NR1H2 | LXRβ | NM_009473 | Mm00437265_g1 |
| NR1H3 | LXRα | NM_013839 | Mm00443454_m1 |
| NR1H4 | FXRα | NM_009108 | Mm00436419_m1 |
| NR1H5 | FXRβ | NM_198658 | Mm01308716_m1 |
| NR1I1 | VDR | NM_009504 | Mm00437302_g1 |
| NR1I2 | PXR | NM_010936 | Mm01344139_m1 |
| NR1I3 | CAR | NM_009803 | Mm01283978_m1 |
| NR2A1 | HNF4α | NM_008261 | Mm00433964_m1 |
| NR2A2 | HNF4γ | NM_013920 | Mm00443563_m1 |
| NR2B1 | RXRα | NM_011305 | Mm00441182_m1 |
| NR2B2 | RXRβ | NM_011306 | Mm00441197_gH |
| NR2B3 | RXRγ | NM_009107 | Mm00436410_m1 |
| NR2C1 | TR2 | NM_011629 | Mm00449123_m1 |
| NR2C2 | TR4 | NM_011630 | Mm01182440_m1 |
| NR2E1 | TLX | NM_152229 | Mm00455855_m1 |
| NR2E3 | PNR | NM_013708 | Mm00443299_m1 |
| NR2F1 | COUP-TFI | NM_010151 | Mm00657937_m1 |
| NR2F2 | COUP-TFII | NM_009697 | Mm00772789_m1 |
| NR2F6 | COUP-TFIII | NM_010150 | Mm00438762_m1 |
| NR3A1 | ERα | NM_007956 | Mm00433149_m1 |
| NR3A2 | ERβ | NM_207707 | Mm00599821_m1 |
| NR3B1 | ERRα | NM_007953 | Mm00433143_m1 |
| NR3B2 | ERRβ | NM_011934 | Mm00442411_m1 |
| NR3B3 | ERRγ | NM_011935 | Mm00516269_mH |
| NR3C1 | GR | NM_008173 | Mm00433832_m1 |
| NR3C2 | MR | XM_098332 | Mm01241597_m1 |
| NR3C3 | PR | NM_008829 | Mm00435628_m1 |
| NR3C4 | AR | NM_013476 | Mm00442688_m1 |
| NR4A1 | NGFI-B | NM_010444 | Mm00439358_m1 |
| NR4A2 | NURR1 | NM_013613 | Mm00443056_m1 |
| NR4A3 | NOR1 | NM_015743 | Mm00450074_m1 |
| NR5A1 | SF1 | NM_139051 | Mm00446826_m1 |
| NR5A2 | LRH1 | NM_030676 | Mm00446088_m1 |
| NR6A1 | GCNF | NM_010264 | Mm00599848_m1 |
| NR0B1 | DAX-1 | NM_007430 | Mm00431729_m1 |
| NR0B2 | SHP | NM_011850 | Mm00442278_m1 |
| 18S | 18S | X00686 | Mm03928990_g1 |
